# Gestational Age at Delivery and Special Educational Need: Retrospective Cohort Study of 407,503 Schoolchildren

**DOI:** 10.1371/journal.pmed.1000289

**Published:** 2010-06-08

**Authors:** Daniel F. MacKay, Gordon C. S. Smith, Richard Dobbie, Jill P. Pell

**Affiliations:** 1 Section of Public Health, University of Glasgow, Glasgow, United Kingdom; 2 Department of Obstetrics and Gynaecology, Cambridge University, The Rosie Hospital Cambridge, United Kingdom; 3 Information Services Division, NHS Scotland, Edinburgh, United Kingdom; 4 Section of Public Health, University of Glasgow, Glasgow, United Kingdom; Chinese University of Hong Kong, Hong Kong

## Abstract

**Background:**

Previous studies have demonstrated an association between preterm delivery and increased risk of special educational need (SEN). The aim of our study was to examine the risk of SEN across the full range of gestation.

**Methods and Findings:**

We conducted a population-based, retrospective study by linking school census data on the 407,503 eligible school-aged children resident in 19 Scottish Local Authority areas (total population 3.8 million) to their routine birth data. SEN was recorded in 17,784 (4.9%) children; 1,565 (8.4%) of those born preterm and 16,219 (4.7%) of those born at term. The risk of SEN increased across the whole range of gestation from 40 to 24 wk: 37–39 wk adjusted odds ratio (OR) 1.16, 95% confidence interval (CI) 1.12–1.20; 33–36 wk adjusted OR 1.53, 95% CI 1.43–1.63; 28–32 wk adjusted OR 2.66, 95% CI 2.38–2.97; 24–27 wk adjusted OR 6.92, 95% CI 5.58–8.58. There was no interaction between elective versus spontaneous delivery. Overall, gestation at delivery accounted for 10% of the adjusted population attributable fraction of SEN. Because of their high frequency, early term deliveries (37–39 wk) accounted for 5.5% of cases of SEN compared with preterm deliveries (<37 wk), which accounted for only 3.6% of cases.

**Conclusions:**

Gestation at delivery had a strong, dose-dependent relationship with SEN that was apparent across the whole range of gestation. Because early term delivery is more common than preterm delivery, the former accounts for a higher percentage of SEN cases. Our findings have important implications for clinical practice in relation to the timing of elective delivery.

*Please see later in the article for the Editors' Summary*

## Abstract 2

A retrospective cohort study of 407,503 schoolchildren by Jill Pell and colleagues finds that gestational age at delivery has a dose-dependent relationship with the risk of special educational needs that extends across the full gestational range.

## Editors' Summary

**Background:**

Most pregnancies last around 40 weeks, but the number of babies who are born early is increasing. In the US, for example, more than half a million babies a year are now born before 37 weeks of gestation (gestation is the period during which a baby develops in its mother). Sadly, babies born this early (premature babies) are more likely to die than babies delivered between 37 and 42 weeks of gestation (term babies) and many have short- and/or long-term health problems. They are also more likely to have a “special educational need” (SEN) as children, a learning difficulty (for example, dyslexia or autism), or a physical difficulty such as deafness or poor vision that requires special educational help, than term babies. The severity of all the problems associated with being born early depends on the degree of prematurity. Thus, the risk of SEN associated with prematurity declines steadily with advancing gestational age up to 36 weeks. That is, a baby born at 24 weeks of gestation is more likely to have an SEN later in life than one born at 36 weeks of gestation.

**Why Was This Study Done?:**

Early term births (between 37 and 39 weeks of gestation) are also becoming more common, because more mothers are electing to be delivered early for nonmedical reasons (so-called birth scheduling). Unfortunately, it is not known whether early term babies have an increased risk of SEN compared with babies born at 40 weeks, because few studies have looked at SEN in children born between 37 and 39 weeks of gestation. In this retrospective cohort study, the researchers investigate the risk of SEN across the whole spectrum of gestational age at delivery and use their results to calculate the fraction of SEN associated with delivery at different gestational ages—the “population attributable risk.” In a retrospective cohort study, researchers examine the medical records of groups of people who differ in a specific characteristic (in this case, having an SEN) for a particular outcome (in this case, gestational age at birth).

**What Did the Researchers Do and Find?:**

The researchers linked 2005 School Census data (which includes a record of all children with an SEN) for more than 400,000 school-aged children in 19 Scottish Local Authority areas (covering three-quarters of the Scottish population) with routine birth data held in the Scottish Morbidity Record. SEN was recorded for nearly 18,000 children (4.9% of the children). 8.4% of the children who were born preterm and 4.7% of those born at term were recorded as having an SEN. The risk of SEN increased across the gestation range from 40 to 24 weeks. Thus, compared to children born at 40 weeks, children born at 37–39 weeks of gestation were 1.16 times as likely to have an SEN—an odds ratio of 1.16. Children born at 33–36, 28–32, and 24–27 weeks were 1.53, 2.66, and 6.92 times as likely to have an SEN, respectively, as children born at 40 weeks. Although the risk of SEN was much higher in preterm than in early term babies, because many more children were born between 37 and 39 weeks (about a third of babies) than before 37 weeks (one in 20 babies), early term births accounted for 5.5% of cases of SEN, whereas preterm deliveries accounted for only 3.6% of cases.

**What Do These Findings Mean?:**

These findings show that gestational age at delivery strongly affects a child's subsequent risk of having an SEN in a dose-dependent manner across the whole range of gestational age. Furthermore, because early term delivery is much more common than preterm delivery, these findings show that early term delivery is responsible for more cases of SEN than preterm delivery. These findings, which are likely to be accurate because of the large size of the study and its design, have important implications for the timing of elective delivery. They suggest that deliveries should ideally wait until 40 weeks of gestation because even a baby born at 39 weeks—the normal timing for elective deliveries these days—has an increased risk of SEN compared with a baby born a week later.

**Additional Information:**

Please access these Web sites via the online version of this summary at http://dx.doi.org/10.1371/journal.pmed.1000289.

- The March of Dimes, a nonprofit organization for pregnancy and baby health, provides information on preterm birth and an article on why the last weeks of pregnancy count (in English and Spanish)
- The US National Institute of Child Health and Human Development has information on preterm labor and birth and on learning disabilities
- The UK Government provides information for parents on special educational needs
- Medline Plus has links to further information on both premature birth and learning disabilities (in English and Spanish)

## Introduction

Infants delivered preterm are at increased risk of neurodevelopmental problems including impaired intelligence and school performance [1],[2]. Amongst preterm infants there is no evidence of a threshold effect, with the risk declining steadily with advancing gestational age up to 36 wk. However, there is a lack of information on whether the increased risk continues across the early term period (37–39 wk of gestation). This question is of considerable clinical relevance because early term births account for an increasing proportion of deliveries [3], and many of these are elective deliveries.

The aim of our study was to investigate the risk of special educational need (SEN) across the whole spectrum of gestational age at delivery, and to use these results to determine the population attributable risks associated with delivery at different gestational ages.

## Methods

### Data Sources

Under the Special Educational Needs and Disability Act of 2001, both schools and local education authorities in the United Kingdom have a statutory duty to identify, assess, and make provision for children with SEN. The Department of Education defines SEN as a learning difficulty that requires special educational provision. In turn, a learning difficulty is defined as either greater difficulty in learning than a majority of children of the same age, or a disability that prevents or hinders a child from making use of educational facilities of the kind that are generally provided for children of the same age (http://www.teachernet.gov.uk/_doc/3724/SENCodeOfPractice.pdf).

SEN includes both children with learning disabilities (including dyslexia, dyspraxia, autism, Asperger's syndrome, and attention deficit hyperactivity disorder), as well as children with physical disabilities that impact on learning (including some children with hearing, motor, and visual impairments). We used data from the 2005 school census. The school census is undertaken annually in September and the data are provided by head-teachers at each school and collated by their local education authority. The school census covers all schools in Scotland irrespective of their funding source and includes local authority, grant-aided, independent, and self-governing schools. The response rate is 99.8%. It includes all primary and secondary school children. It excludes adults (>19 y of age) who are attending courses held in schools. It covers mainstream schools, special schools, and special classes and units within mainstream schools. Children on long-term illness absence are included. The information is collected at the level of individual pupils and includes a record of need for all children with an SEN. Of the 32 Scottish local education authorities, 19 agreed to participate and provide data from the school census. The participating authorities covered a total population of 3.8 million, equivalent to 74% of the Scottish population (http://www.statistics.gov.uk/STATBASE/Expodata/Spreadsheets/D5966.xls).

The Scottish Morbidity Record (SMR2) collects information on all women discharged from Scottish maternity hospitals, including maternal and infant characteristics, clinical management, and obstetric complications. The SMR2 is subjected to regular quality assurance checks and has been more than 99% complete since the late 1970s [4]. A quality assurance exercise performed in 1997 compared a 5% sample of SMR2 returns (*n* = 1,414) to case records and demonstrated that all of the fields used in our study had less than 2% errors, with the exception of maternal height (4.4%), estimated gestation (5.6%), and induction of labour (6.4%) (Jim Chalmers, Information Services Division, National Health Service, Edinburgh, Scotland, written communication, April 2001). In the SMR2, gestational age at birth is defined as completed weeks of gestation on the basis of the estimated date of delivery recorded in each woman's clinical record. Gestational age has been confirmed by ultrasound in the first half of pregnancy in more than 95% of women in the United Kingdom since the early 1990s [5]. Previous miscarriage was defined as previous delivery of a conceptus, showing no signs of life before 24 wk gestation, excluding therapeutic abortions. Previous therapeutic abortion was defined as previous therapeutic termination of pregnancy, by any means, prior to 24 wk gestation.

The school census data were linked, via birth certificate data, to the relevant SMR2 record to provide individual-level obstetric data. We excluded individuals who were aged <4 y or >19 y at the time of the school census, and births where the maternal height was recorded as less than 100 cm or greater than 200 cm, the birth weight was recorded as less than 400 g or greater than 5,000 g, or the gestation at delivery was recorded as less than 24 wk or greater than 43 wk. We also excluded multiple births because the SMR2 record does not record infant name. Therefore, in the case of multiple births, we could not ensure that the school census record was linked to the correct infant. Permission to access, link, and analyse these data was granted by both the South-East of Scotland Multi-Centre Research Ethics Committee and the Scottish Privacy Advisory Committee.

### Statistical Analysis

Continuous variables were summarised by the median and interquartile range (IQR). Univariate comparisons between groups were performed using the Kruskal-Wallis test, Chi square test, and Cuzick's test for trend [6] for continuous, categorical, and ordinal data, respectively. The *p*-values for all hypothesis tests were two-sided and statistical significance was assumed at *p*<0.05. The associations between obstetric factors and the risk of SEN were analysed using univariate and multivariable logistic regression and presented as odds ratios. The covariates included in the multivariable analysis were infant sex, maternal age and height, marital status, parity, birth-weight centile, induction of labour, mode of delivery, year of delivery, previous spontaneous and therapeutic deliveries, and 5-min Apgar score.

Missing values for maternal height, deprivation, and maternal age were created using multiple imputation by chained equations through the use of the ICE module available in STATA [7]. Variables included in the imputation included all covariates in the final model and the outcome variable. Five imputed datasets were created and a sensitivity analysis of complete cases with the imputed datasets was conducted. Gestation- and sex-specific birth-weight centiles were calculated. Year of delivery of the child was included as a covariate. Years 1980 to 1987 and years 2000 to 2003 were aggregated because of the small numbers of children born in these years.

Goodness of fit was assessed using a plot of observed versus expected values as well as residual and influence plots. These tests showed that the model was an adequate fit to the data. The area under the ROC curve was equal to 67.6%. Adjusted population attributable fractions [8] were estimated using individuals with complete data to determine what proportion of SEN cases were potentially explained by gestation at delivery. All statistical analyses were undertaken using STATA V10.1 (Stata Corporation).

## Results

Of the 514,118 children included in the school census, 93,340 (18.2%) could not be linked to their obstetric data and 4,998 (5.3%) of these had a record of SEN. Of the 420,778 (81.8%) who were successfully linked to their obstetric data, 13,275 were ineligible for inclusion because they fulfilled one or more exclusion criteria. Of the remaining 407,503 children, 362,688 (89.0%) children had complete data on all variables and, of these, 17,784 (4.9%) had a record of SEN. The children had a median age of 12 y, with no significant difference according to the presence or absence of SEN.

Overall, 184,260 (50.8%) infants were male, 18,527 (5.0%) were born preterm (<37 wk gestation), 58,611 (16.2%) were delivered by cesarean section, and 10,404 (2.8%) had a 5-min Apgar score less than eight. The median birth weight was 3,400 g (IQR 3,060–3,740). The mothers had a median age of 28 y (IQR 24–31) at delivery and a median height of 162 cm (IQR 157–166). Among the mothers, 203,114 (56.0%) were multiparous, 115,356 (31.8%) were unmarried at the time of delivery, and 12,579 (3.5%) suffered pre-eclampsia. 67,181 (18.5%) had a history of spontaneous abortion and 35,483 (9.8%) had a history of therapeutic abortion. Table 1 presents a breakdown of maternal and pregnancy characteristics according to whether or not the child had an SEN (Table 1).

**Table 1:** Pregnancy characteristics of schoolchildren according to whether they have special educational needs.

Pregnancy Characteristics	No Special Educational Need, *n* = 387,682	Special Educational Need, *n* = 19,821	*p*-Value^a^
**Gestation at delivery (wk)**	***n*** ** (%)**	***n*** ** (%)**	
24–27	335 (0.09)	140 (0.7)	<0.001
28–32	3,006 (0.8)	443 (2.2)	
33–36	16,754 (4.3)	1,281 (6.5)	
37	18,617 (4.8)	1,217 (6.1)	
38	48,810 (12.6)	2,759 (13.9)	
39	77,217 (19.9)	3,848 (19.4)	
40	125,067 (32.3)	5,731 (28.9)	
41	81,607 (21.1)	3,530 (17.8)	
42	15,936 (4.1)	850 (4.3)	
43	333 (0.08)	22 (0.11)	
Missing	0	0	
**Sex**			
Female	194,648 (50.2)	5,934 (29.9)	<0.001
Male	193,034 (49.8)	13,887 (70.1)	
Missing	0	0	
**Parity**			
Nulliparous	172,935 (44.6)	7,473 (37.7)	<0.001
Multiparous	214,747 (55.7)	12,348 (62.3)	
Missing	0	0	
**Induced**			
No	296,449 (76.5)	15,307 (77.2)	0.014
Yes	91,233 (23.5)	4,514 (22.8)	
Missing	0	0	
**Previous spontaneous abortions**			
0	316,005 (81.5)	15,762 (79.5)	<0.001
1	56,360 (14.5)	3,093 (15.6)	
≥2	15,317 (4.0)	966 (4.9)	
Missing	0	0	
**Previous therapeutic abortions**			
0	349,982 (90.3)	17,602 (88.8)	<0.001
1	33,215 (8.6)	1,918 (9.7)	
≥2	4,485 (1.1)	301 (1.5)	
Missing	0	0	
**Deprivation quintiles**			
1 (affluent)	63,073 (16.3)	2,731 (13.8)	<0.001
2	65,094 (16.9)	3,063 (15.5)	
3	78,692 (20.4)	4,029 (20.4)	
4	80,406 (20.8)	4,317 (21.9)	
5 (deprived)	98,837 (25.6)	5,611 (28.4)	
Missing	1,580	70	
**Marital status**			
Married	262,980 (67.8)	11,911 (60.1)	<0.001
Not married	124,702 (32.2)	7,910 (39.9)	
Missing	0	0	
**Birth-weight centiles**			
1–3	11,447 (3.0)	1,084 (5.5)	<0.001
4–10	27,037 (7.0)	1,865 (9.4)	
11–20	39,238 (10.1)	2,334 (11.8)	
21–80	233,002 (60.1)	11,110 (56.1)	
81–90	38,496 (9.9)	1,687 (8.5)	
91–97	27,094 (7.0)	1,230 (6.2)	
98–100	11,368 (2.9)	511 (2.6)	
Missing	0	0	
**Pre-eclampsia**			
No	373,782 (96.4)	19,023 (96.0)	0.001
Yes	13,900 (3.6)	798 (4.0)	
Missing	0	0	
**5-min Apgar score**			
0–3	2,647 (0.7)	303 (1.5)	<0.001
4–7	8,144 (2.1)	782 (4.0)	
8–10	376,891 (97.2)	18,736 (94.5)	
Missing	0	0	
**Mode of delivery**			
Vaginal, cephalic delivery	269,294 (69.5)	13,658 (68.9)	<0.001
Assisted vaginal delivery	54,346 (14.0)	2,358 (11.9)	
Breech delivery	1,601 (0.4)	145 (0.70)	
Cesarean section (elective)	23,846 (6.2)	1,343 (6.8)	
Cesarean section (emergency, prelabour)	10,043 (2.6)	782 (4.0)	
Cesarean section (emergency, postlabour)	28,534 (7.4)	1,535 (7.7)	
Missing	0	0	
**Median maternal age (y) (IQR)**	27 (23–31)	28 (24–31)	<0.001
Missing	1	0	
**Median maternal height (cm) (IQR)**	161 (157–165)	162 (157–166)	<0.001
Missing	41,328	1,967	

a Kruskal-Wallis test for continuous data; Cuzick's nonparametric test for trend for ordinal data; χ^2^ test for categorical data.

**Figure 1:**
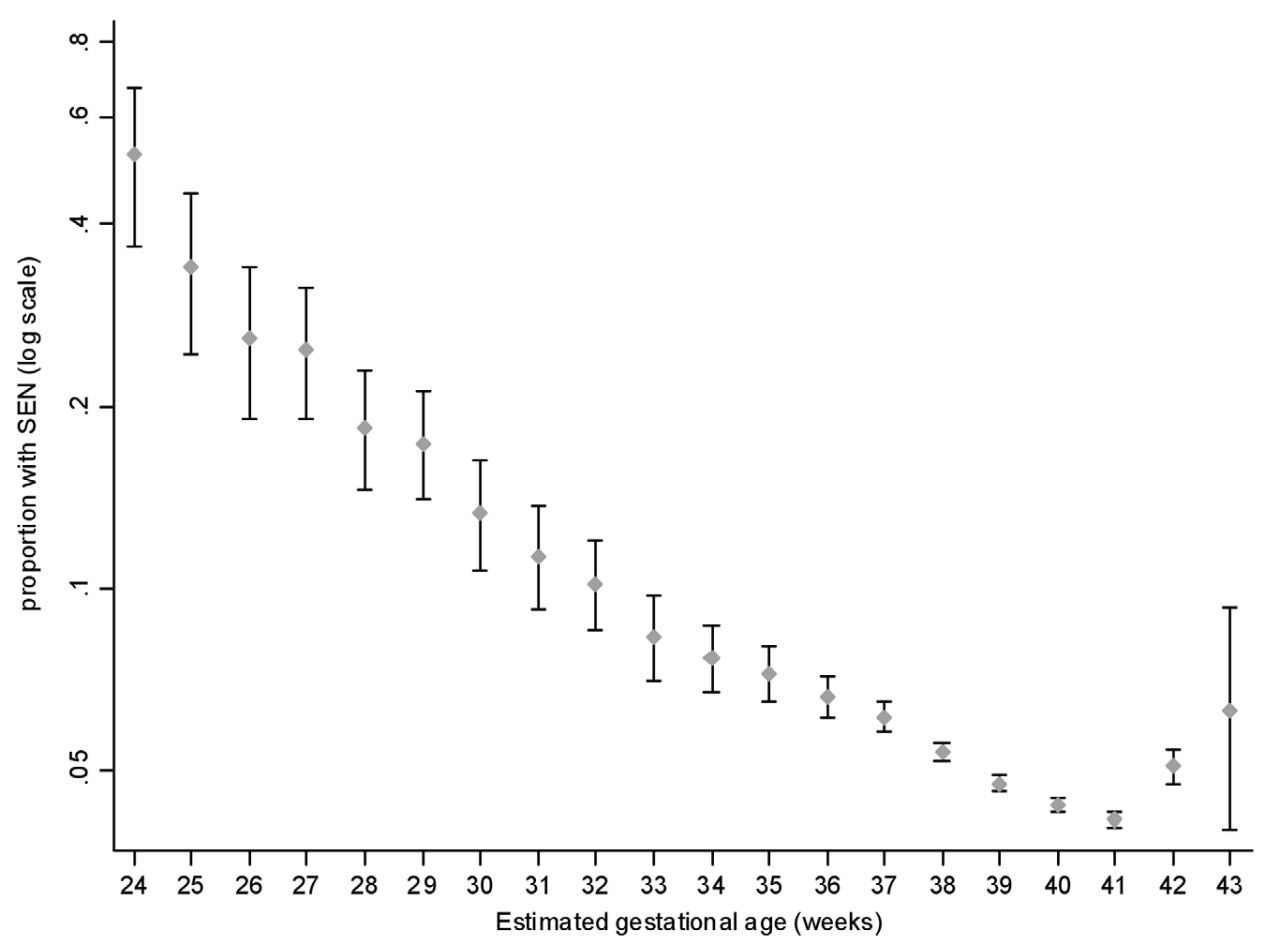
Prevalence of special educational need by gestation at delivery.

Low birth weight (<2,500 g) was associated with an increased risk of subsequent SEN (unadjusted odds ratio [OR] 2.22, 95% confidence interval [CI] 2.10–2.33, *p*<0.001). This result was explained by associations with both preterm delivery and low birth-weight centile (Table 2). The relationship between SEN and gestation at delivery was reverse J-shaped (Figure 1). The reverse J-shaped relationship was tested using a multivariable restricted cubic spline, which confirmed that the effect of gestational age was robust to the inclusion of the covariates. There was a very strong association with extreme preterm delivery (24–27 wk: adjusted OR 6.92, 95% CI 5.58–8.58, *p*<0.001) (Table 2). The risk steadily declined with increasing gestational age up to 40–41 wk, but then increased among those who delivered post-dates (42 wk; adjusted OR 1.16, 95% CI 1.08–1.25, *p*<0.001). Compared with infants born at 40 wk, those delivered early term (37–39 wk) were at increased risk of SEN (adjusted OR 1.16, 95% CI 1.12–1.20, *p*<0.001). The risk decreased across the early term range, from 39 to 37 wk, but nonetheless was significantly increased among children born at 39 wk of gestation (adjusted OR 1.09, 95% CI 1.04–1.14, *p*<0.001). There was no statistically significant interaction between gestational age and elective versus spontaneous delivery. The results were very similar when we re-ran the logistic regression models without imputation of missing values. Maternal smoking status was only recorded on SMR2 from 1992 onwards. When we re-ran the models for this subgroup of children including maternal smoking data, the J-shaped relationship remained and the strength of association between gestational age at delivery and risk of SEN was barely altered (37–39 wk: adjusted OR 1.12, 95% CI 1.07–1.18, *p*<0.001). The population attributable fraction of SEN accounted for by gestation as a whole was 10.0% (Table 3). Preterm delivery had a population attributable fraction of 3.6% compared with 5.5% for early term deliveries (Table 3). Text S1 contains a sensitivity analysis showing that the findings could not be explained by bias due to missing data.

**Table 2:** Univariate and multivariable logistic regression analysis of the association between pregnancy characteristics and risk of special educational needs in schoolchildren.

Pregnancy Characteristics	Univariate		Multivariable^a^	
	OR (95% CI)	*p*-Value	OR (95% CI)	*p*-Value
**Gestation at delivery (wk)**				
24–27	9.14 (7.53–11.18)	<0.001	6.92 (5.58–8.58)	0.001
28–32	3.21 (2.90–3.57)	<0.001	2.66 (2.38–2.97)	<0.001
33–36	1.67 (1.57–1.78)	<0.001	1.53 (1.43–1.63)	<0.001
37	1.43 (1.34–1.52)	<0.001	1.36 (1.27–1.45)	<0.001
38	1.24 (1.18–1.29)	<0.001	1.19 (1.14–1.25)	<0.001
39	1.09 (1.05–1.14)	<0.001	1.09 (1.04–1.14)	<0.001
40	1.00		1.00	
41	0.95 (0.90–0.98)	0.010	0.97 (0.93–1.01)	0.188
42	1.17 (1.09–1.26)	<0.001	1.16 (1.08–1.25)	<0.001
43	1.46 (0.95–2.25)	0.085	1.35 (0.87–2.09)	0.180
***p*** ** for trend**		<0.001		<0.001
**Sex**				
Female	1.00		1.00	
Male	2.36 (2.29–2.43)	<0.001	2.36 (2.28–2.43)	<0.001
**Maternal age (y)**				
**<20**	1.37 (1.30–1.45)	<0.001	1.42 (1.34–1.51)	<0.001
20–24	1.26 (1.21–1.31)	<0.001	1.21 (1.16–1.26)	<0.001
25–29	1.00		1.00	
30–34	0.92 (0.89–0.96)	<0.001	0.91 (0.88–0.95)	<0.001
35–39	0.98 (0.93–1.04)	0.490	0.93 (0.88–0.98)	0.012
>39	1.31 (1.17–1.48)	<0.001	1.19 (1.05–1.34)	0.005
***p*** ** for trend**		<0.001		<0.001
**Maternal height (cm)**				
<150	1.55 (1.42–1.69)	<0.001	1.25 (1.14–1.37)	<0.001
150–154	1.22 (1.16–1.28)	<0.001	1.08 (1.03–1.14)	0.003
155–159	1.05 (1.01–1.09)	0.017	0.99 (0.95–1.03)	0.609
160–164	1.00		1.00	
165–169	0.95 (0.91–0.99)	0.010	1.01 (0.97–1.06)	0.566
170–174	0.94 (0.89–1.00)	0.035	1.07 (1.00–1.13)	0.038
>174	0.92 (0.83–1.01)	0.071	1.09 (0.97–1.19)	0.154
***p*** ** for trend**		<0.001		0.186
**Marital status**				
Married	1.00		1.00	
Not married	1.40 (1.36–1.44)	<0.001	1.36 (1.31–1.40)	<0.001
**Deprivation**				
1 (affluent)	0.84 (0.80–0.89)	<0.001	0.95 (0.91–1.00)	0.100
2	0.92 (0.88–0.97)	0.001	0.97 (0.93–1.02)	0.378
3	1.00		1.00	
4	1.05 (1.01–1.10)	0.027	0.98 (0.94–1.03)	0.462
5 (deprived)	1.11 (1.07–1.16)	<0.001	0.94 (0.90–0.98)	0.002
***p*** ** for trend**		<0.001		0.343
**Parity**				
Nulliparous	1.00		1.00	
Multiparous	1.33 (1.29–1.37)	<0.001	1.61 (1.55–1.66)	<0.001
**Previous spontaneous abortion**				
0	1.00		1.00	
1	1.10 (1.06–1.15)	<0.001	1.09 (1.05–1.14)	<0.001
≥2	1.27 (1.18–1.35)	<0.001	1.21 (1.13–1.30)	<0.001
***p*** ** for trend**		<0.001		<0.001
**Previous therapeutic abortion**				
0	1.00		1.00	
1	1.15 (1.09–1.20)	<0.001	1.10 (1.04–1.15)	<0.001
≥2	1.34 (1.19–1.50)	<0.001	1.26 (1.12–1.42)	0.001
***p*** ** for trend**		<0.001		<0.001
**Birth-weight centiles**				
1–3	1.99 (1.86–2.12)	<0.001	1.95 (1.82–2.08)	<0.001
4–10	1.45 (1.38–1.52)	<0.001	1.43 (1.35–1.50)	<0.001
11–20	1.25 (1.17–1.31)	<0.001	1.24 (1.18–1.30)	<0.001
21–80	1.00		1.00	
81–90	0.92 (0.88–0.97)	0.003	0.93 (0.88–0.98)	0.005
91–97	0.95 (0.90–1.01)	0.103	0.95 (0.89–1.01)	0.077
98–100	0.95 (0.87–1.04)	0.248	0.93 (0.85–1.02)	0.130
***p*** ** for trend**		<0.001		<0.001
**Pre-eclampsia**				
None	1.00		1.00	
Yes	1.14 (1.06–1.22)	0.001	0.99 (0.92–1.07)	0.829
**Induced**				
No	1.0		1.00	
Yes	0.96 (0.93–0.99)	0.016	1.03 (0.99–1.07)	0.126
**Mode of delivery**				
Vaginal, cephalic delivery	1.00		1.00	
Assisted vaginal delivery	0.86 (0.82–0.89)	<0.001	1.04 (0.99–1.09)	0.132
Breech delivery	1.77 (1.49–2.10)	<0.001	1.22 (1.02–1.47)	0.031
Cesarean section (elective)	1.10 (1.04–1.17)	0.001	1.06 (0.99–1.13)	0.075
Cesarean section (emergency, prelabour)	1.54 (1.43–1.66)	<0.001	1.19 (1.10–1.29)	<0.001
Cesarean section (emergency, postlabour)	1.06 (1.00–1.12)	0.034	1.13 (1.07–1.20)	<0.001
***p*** ** for trend**		<0.001		<0.001
**5-min APGAR score**				
0–3	2.30 (2.04–2.59)	<0.001	1.66 (1.46–1.88)	<0.001
4–7	1.94 (1.80–2.09)	<0.001	1.55 (1.43–1.67)	<0.001
8–10	1.00		1.00	
***p*** ** for trend**		<0.001		<0.001

a Also adjusted for year of delivery.

**Table 3:** Population attributable fractions for special educational needs due to gestational age at delivery (wk).

Gestation at Delivery (wk)	Attributable Fraction^a^	95% CI
**24–27**	0.005	(0.004–0.006)
**28–32**	0.011	(0.010–0.013)
**33–36**	0.020	(0.017–0.024)
**37**	0.016	(0.012–0.020)
**38**	0.020	(0.016–0.028)
**39**	0.019	(0.012–0.027)
**42**	0.007	(0.003–0.010)
**43**	0.000	(0.000–0.000)
**Total**	0.100	(0.007–0.129)

a Adjusted for infant sex, maternal age and height, marital status, parity, birth-weight centile, induction of labour, mode of delivery, year of delivery, previous spontaneous and therapeutic deliveries, and 5-min Apgar score.

## Discussion

Our study demonstrated a strong trend of decreasing risk of SEN with advancing gestational age at birth. The key finding of the present analysis is that this trend continued across gestational ages classified as term. Although the risk of SEN was highest among infants who were delivered preterm (<37 wk gestation), these accounted for only 5.1% of deliveries. Therefore, only a relatively small proportion of SEN (3.5%) could be attributed to preterm delivery. By contrast, 39.6% of infants were delivered between 37 and 39 wk gestation. Therefore, whilst these early term infants had only a moderately increased risk, 5.3% of SEN cases could be attributed to early term delivery.

The association between gestational age and risk of SEN was strong, consistent, and demonstrated a dose response. Historically, preterm delivery has been the main focus of research and clinical efforts because of the high risk to the individual infant. The neurodevelopmental sequelae of early term deliveries may be too subtle to be observed prior to commencing school. Nonetheless, our study suggests that, at a population level, they are a contributor to SEN. In the United States, there has been an absolute increase of 8.9% in deliveries of early term infants over the past decade, with all of this increase occurring among elective deliveries, largely due to an increase in cesarean section upon request [3]. In Scotland, between 1980 and 2005, there has been a 2.0% absolute increase in the proportion of live-born infants delivered preterm (from 5.4% to 7.4% of all deliveries) compared with a 6.2% absolute increase in the proportion of live-born infants delivered at early term (from 33.2% to 39.4%) (unpublished SMR2 data). Therefore, the population impact of SEN in this subgroup of infants is likely to increase.

Previous studies have shown that preterm delivery is associated with neurodevelopmental problems including developmental delay, learning and language difficulties, impaired cognitive function, and behavioural problems [9]–[14]. Studies suggest that whereas all preterm infants are at increased risk, the risk increases across the preterm spectrum from late preterm delivery (34–36 wk), through very preterm delivery (28–34 wk), to extreme preterm delivery (<28 wk) [15],[16]. The percentage of infants born early term (37–39 wk) has risen dramatically over the past decade [3]. In spite of this increase, there is a relative paucity of research into the risk of SEN in this group of children. Our study did not demonstrate any evidence of a threshold effect at 37 wk. Therefore, the tendency of most previous studies to treat gestation as a binary factor (preterm versus term) has masked a dose-effect across the whole range of gestation.

A small number of studies have examined the relationship between gestational age, rather than preterm delivery, and either school performance or intelligence scores. In a study of 317,761 male, Norwegian conscripts, mean intelligence score increased from 28 to 40–41 wk gestation [15]. However, individuals with significant intellectual impairment were exempt from conscription and were, therefore, excluded from the study. More recently, a study of 13,824 children in Belarus confirmed an increase in intelligence score from 37 to 40 wk gestation [16]. Small reductions in cognitive tests and IQ do not necessarily equate to detectable differences in school performance or children's ability to function in society. In a study of 873 Swedish children, Lagerstrom et al. found no significant association between gestational age and either school performance or intelligence scores at 13 y of age [17]. In a study of 5,319 Danish children, parents reported their child's performance at school at 10 y of age [18]. Compared with children delivered at 39–40 wk gestation, those delivered at 37–38 wk had a higher risk of reading and spelling difficulties. No association was found with arithmetic difficulties. The study excluded children born at less than 33 wk gestation and was underpowered to examine the effect of delivery prior to 37 wk. Therefore, the investigators were unable to determine whether there was a significant trend across the spectrum of gestational age. In the Bavarian Longitudinal Study, IQ decreased by around 2.5 points for each week of gestation from 32 to 27 wk [19].

Our study demonstrated that the risk of SEN was lowest at 40–41 wk gestation and rose beyond this. The effect of delivery post-dates has been ignored in most previous studies. Among Norwegian conscripts, intelligence scores fell from 41 to 44 wk gestation [15] and, in a study of 86,630 children born in 1950–1954, verbal reasoning scores in the 11 plus examination were lower among those delivered beyond 40 wk gestation [20]. Similarly, among children in Belarus, intelligence score fell from 40 to 43 wk of gestation [16]. In contrast, a longitudinal study of 575 normal birth-weight infants demonstrated significantly higher IQ scores at 8–10 y among those delivered at >40 wk compared those delivered at 38–40 wk [21].

Our study has a number of strengths. It is the largest study of its kind and the first unselected, population-based study to examine risk over the whole gestational spectrum. Being based on a national population register, it is not subject to selection bias. The obstetric and SEN data were obtained from routine data sources obviating possible recall, response, or ascertainment bias. Our ascertainment of SEN included all school-age children irrespective of type of school and was, therefore, representative of the population as a whole. Because Scotland has a stable population and high quality routine data sources, a very high percentage of pupils could be linked to their birth records. The obstetric register is detailed and accurate enabling adjustment for a wide range of possible confounding factors. A number of previous studies have examined birth weight, rather than gestation. Although mean birth weight is lower among preterm infants, individual low birth weight is a poor proxy of gestation since it is also a marker of intra-uterine growth restriction. Furthermore, there is an interaction between absolute birth weight and gestational age, such that low birth weight is a risk factor for intellectual impairment among term infants but not preterm infants [14],[15],[17]. Therefore, a specific strength of our study is that we measured gestation and adjusted for sex- and gestation-specific birth-weight centile, rather than using absolute birth weight. Wiener et al. restricted their analysis to infants of normal birth weight and demonstrated lower IQ scores at 8–10 y of age among those born preterm compared with those born at term [21].

Early term deliveries have increased over time. The normal timing of elective delivery has changed over time from 37 towards 39 wk. 75% of deliveries at 39 wk are elective (unpublished SMR2 data). However, our data suggest that even at 39 wk infants are at increased risk of SEN and account for 1.7% of all SEN cases. These findings have implications for clinical practice in relation to both the decision to undertake elective delivery and the timing of elective deliveries.

## Supporting Information

Text S1 Sensitivity analysis.
(0.04 MB DOC)

## Abbreviations

CI: confidence interval
OR: odds ratio
SEN: special educational need
SMR2: Scottish Morbidity Record
