# Supplementary material for: Gestational Age at Delivery and Special Educational Need: Retrospective Cohort Study of 407,503 Schoolchildren
Source: PLoS Med. 2010 Jun 8;7(6):e1000289. doi: 10.1371/journal.pmed.1000289 (PMC2882432; doi:10.1371/journal.pmed.1000289)
Supplement: Text S1 — Sensitivity analysis. (0.04 MB DOC) [file pmed.1000289.s001.doc]

**Text S1. Sensitivity analysis**

We know that there are 93,340 children with unlinked census records of whom 4,988 have SEN and 88,352 do not have SEN.

The overall prevalence of preterm delivery among linked children is equivalent to that in the whole population (SMR2). Therefore, the overall prevalence of preterm should also be the same among unlinked children (5.4%). Let us consider the worse case scenario in which there is absolutely no association between gestation and SEN among unlinked children. Therefore, the prevalence of preterm will be the same in both unlinked children with SEN and unlinked children without SEN.

| Unlinked | No SEN | SEN |  |
| --- | --- | --- | --- |
| Preterm | 4,771 | 269 | 5,040 |
| Term | 83,581 | 4,719 | 88,300 |
|  | 88,352 | 4,988 | 93,340 |

Unadjusted RR = 269 x 88,352 / 5,040 x 4,771 = 1.00

We know that among linked children (prior to application of SMR2 based exclusions) …

| Linked | No SEN | SEN |  |
| --- | --- | --- | --- |
| Preterm | 24,910 | 2,295 | 27,205 |
| Term | 375,180 | 18,393 | 393,573 |
|  | 400,000 | 20,688 | 420,778 |

Unadjusted RR = 2,295 x 393,572 / 27,205 x 18,393 = 1.81

Χ^2^ = 770.60

Using 95% CI = RR^(1+1.96/χ)^ and RR^(1-1.96/χ)^

95% CI = (1.74-1.89)

Therefore if we had been able to include the unlinked children with the linked children in the analysis we would have obtained ….

| Unlinked and linked | No SEN | SEN |  |
| --- | --- | --- | --- |
| Preterm | 29,681 | 2,564 | 32,245 |
| Term | 458,761 | 23,112 | 481,873 |
|  | 488,352 | 25,676 | 514,118 |

Unadjusted RR = 2,564 x 481,873 / 32,245 x 23,112 = 1.66

Χ^2^ = 634.17

Using 95% CI = RR^(1+1.96/χ)^ and RR^(1-1.96/χ)^

95% CI = (1.60-1.73)

In conclusion, in this worse case scenario the existing unadjusted RR of 1.81 (95% CI 1.74-1.89) should really have been 1.66 (95% CI 1.60-1.73) – ie there would still have been a significant association between preterm delivery and SEN had all children been linked successfully

(NB we did not apply the exclusion criteria to the linked children, as we are unable to apply them to the unlinked children and had to treat both groups the same.)
